# Probing language identity encoded in pre-trained multilingual models: a typological view

**DOI:** 10.7717/peerj-cs.899

**Published:** 2022-03-15

**Authors:** Jianyu Zheng, Ying Liu

**Affiliations:** Department of Chinese Language and Literature, Tsinghua University, Beijing, China

**Keywords:** Language identity, Pre-trained model, Language model, Typology, Multilingual model

## Abstract

Pre-trained multilingual models have been extensively used in cross-lingual information processing tasks. Existing work focuses on improving the transferring performance of pre-trained multilingual models but ignores the linguistic properties that models preserve at encoding time—“language identity”. We investigated the capability of state-of-the-art pre-trained multilingual models (mBERT, XLM, XLM-R) to preserve language identity through language typology. We explored model differences and variations in terms of languages, typological features, and internal hidden layers. We found the order of ability in preserving language identity of whole model and each of its hidden layers is: mBERT > XLM-R > XLM. Furthermore, all three models capture morphological, lexical, word order and syntactic features well, but perform poorly on nominal and verbal features. Finally, our results show that the ability of XLM-R and XLM remains stable across layers, but the ability of mBERT fluctuates severely. Our findings summarize the ability of each pre-trained multilingual model and its hidden layer to store language identity and typological features. It provides insights for later researchers in processing cross-lingual information.

## Introduction

Pre-trained language models such as BERT (Devlin et al., 2019) and XLNET (Yang et al., 2019) have been widely used in natural language processing tasks in high-resource languages recently. Such tasks include dialogue systems (Wu et al., 2020), text classification (Sun et al., 2019), and reading comprehension (Xu et al., 2019), *etc.* However, for low-resource languages, these models fail to transfer the knowledge from high-resource languages to low-resource languages due to the limited corpus and lack of bilingual alignment data (Cruz & Cheng, 2019). To extend the profits of pre-trained models to low-resource languages, pre-trained multilingual models have been developed (Conneau et al., 2020; Devlin et al., 2019; Lample & Conneau, 2019). These pre-trained multilingual models attempt to map words from different languages to a shared vector space, and extract the semantic relations between words across languages. Depending on the training objectives, pre-trained multilingual models can be categorized into unsupervised models and supervised models. Supervised models, such as XLM (Lample & Conneau, 2019), are trained using bilingual parallel data with a cross-lingual objective; unsupervised models, such as mBERT (Devlin et al., 2019) and XLM-R (Conneau et al., 2020) use different monolingual text as the training corpus each time. Nevertheless, both models have been widely used (Hu et al., 2020).

Although the pre-trained multilingual models have demonstrated superior performance in cross-lingual tasks, there still exists a gap compared to the aligned bilingual word vectors (Cao, Kitaev & Klein, 2019). Researchers argue that although pre-trained multilingual models can learn common patterns across languages, some unique features of each language are also retained (Zhao et al., 2021). These linguistic properties reflect the identity information of this language, but affect the transferability of the model. To bridge the gap, various “language agnostic” operations have been proposed to eliminate language identities (Cao, Kitaev & Klein, 2019; Zhao et al., 2021). However, previous work has paid little attention to the language identity encoded by the model. Therefore, we seek to find the measures of detecting the language identity.

Language typology focuses on the classification of languages based on their structural features (Ferguson, 1959). In language typology, if there is a certain difference between languages, this difference is not casual, but restricted. Language typology is more concerned with the degree of the difference between languages. Due to the limited nature of the difference, it actually becomes meaningful to classify languages with respect to language typology (Velupillai, 2012; Bakker et al., 2009). Its aim is to describe and explain the common properties and the structural diversity of the world’s languages (Ferguson, 1959). Language typology describes a specific language from lexicons, word order and syntax, *etc* (Plungyan, 2011). Therefore, exploring the ability of pre-trained multilingual models to preserve and identify language identity from a typological perspective is feasible.

Inspired by this idea, the characteristics of a certain language can be described from the perspective of typology. If the pre-trained multilingual encoder recognizes the typological features of this language more accurately, the characteristics of the language are better preserved.

A certain language is described by many typological features from different areas. So the average of the prediction values on these typological features can be seen as a kind of prediction indicator, which reflects the ability of the encoder to preserve and identify the language identity. Specifically, we collected a series of typological features, including lexicon, word order, syntax and clauses, *etc.* We trained a simple classifier and predicted the typological feature labels of sentences in different languages after they are represented by each pre-trained multilingual encoder. The ability of the model to preserve and identify language identity can be demonstrated in terms of the prediction accuracy. The primary contributions of this work are four-fold.

1) We found that mBERT preserves language identities the best, XLM-R the second best, and XLM the worst.
2) The ability of each model to encode different languages and capture different typological features is different.
3) Across the layers, XLM and XLM-R are relatively stable, while mBERT varies greatly due to the influence of different languages and typological features.
4) In terms of Chinese, all the three models can capture the lexical and morphological features of Chinese well, but they cannot encode the syntactic, word order, and nominal categories effectively.

The abilities of the whole model and its layers to encode Chinese language identities are below the average of the sample languages. We hope that our work can provide support and inspiration for the subsequent researchers in cross-lingual information processing. The paper is organized as follows. The ‘Related Work’ is introduced first. Then our probing method, including the involved multilingual encoders, is described in ‘Method’. In ‘Experiment’, we first introduce the experimental preparation, such as typological features and dataset, and then display the experimental results and the detailed analysis from two perspectives: language level and typology level. After that, the probing across layers is carried out, and the results from language and typology level are analyzed (‘Probing Across Layers’). Finally, a case study about the Chinese language is provided in ’Case Study’.

### Related Work

### Language identity in pre-trained multilingual models

Pre-trained multilingual models have been widely used in many NLP tasks, such as machine translation (Zhao et al., 2020), information extraction (Jiang et al., 2020), and reading comprehension (Kuratov & Arkhipov, 2019). Although many pre-trained multilingual models are trained on monolingual data, these models can still achieve good performance in many cross-lingual downstream tasks. This suggests that these models can capture universal patterns across languages (Pires, Schlinger & Garrette, 2019). Libovicky pointed out that although the models perform well on the zero-shot cross-lingual tasks, the identity information of the language is still largely preserved (Libovický, Rosa & Fraser, 2020), which affects further improvements in transfer. A number of ”language agnostic” operations have been proposed to eliminate identity differences among languages. For example, Cao, Kitaev & Klein (2019) adopted a series of contextual alignments to improve the model performance in cross-lingual lexical inference (XNLI) and word retrieval. Zhao et al. (2021)) normalized the multilingual representations by re-mapping, batch normalization and pre-processing the input text to improve the models’ performance on downstream tasks such as XNLI and reference-free MT evaluation (RFEval). Previous works have tended to eliminate language identities, but very few studies have focused on the language identity encoded by the model and the way to detect it.

### Language typology in NLP

Language typology classifies languages according to linguistic properties (Bjerva & Augenstein, 2018). Much previous work has investigated pre-trained multilingual models through language typology. Pires, Schlinger & Garrette (2019) probed the generalization capacity of mBERT among languages by two typological features, subject-object-verb order and adjective-noun order. (Choenni & Shutova (2020) studied the ability of pre-trained multilingual models in encoding language typology. Gerz et al. (2018) explored the influence of typology on pre-trained multilingual models’ transferring abilities across languages by means of perplexity scores.

We draw inspiration from the work of Choenni & Shutova (2020), where they explored how pre-trained multilingual encoders capture typological properties. However, they did not explore the capability of each model in preserving language characteristics based on the typological information of that language. In addition, there are some strong assumptions in their work. For example, they assume that each sentence from a certain language possess all typological information of this language. In fact, this might be because when an example sentence is input into the pre-trained multilingual model, the language identity of the sentence could be identified. The major differences between our work and Choenni and Shutova’s work lie in several aspects. First, we probe language identity encoded in pre-trained multilingual models (mBERT, XLM, XLM-R) with respect of typology. The experiment involves more languages, but not always the same features, which makes the experimental results more reliable; Second, we investigated the differences of models’ abilities to preserve and identify language identity from each hidden layer, language family and language group; Finally, we took Chinese as an example and compared it with the average of sample languages. We analyzed and interpreted the results of typological features in Chinese language predicted by the three models from the perspective of typology.

### Method

### Pre-trained multilingual encoders

*Multilingual BERT* (Devlin et al., 2019) is a multilingual version of the BERT model. It is trained on Wikipedia corpus with 12 layers and 768 hidden states. The vocabulary size is 110k, and it has a shared WordPiece vocabulary for tokenization. It takes the Masked Language Modeling (MLM) and the Next Sentence Prediction (NSP) as tasks and supports 104 languages.

*Cross-lingual Language Model* is another transformer-based multilingual language model (Pires, Schlinger & Garrette, 2019), which is trained on Wikipedia corpus. It has 12 layers and 1,024 hidden states. The vocabulary size is 95k, and it uses the Byte Pair Encoding (BPE) for wordpiece. It takes Masked Language Modeling (MLM) and Translation Language Modeling (TLM) as tasks. Cross-lingual Language Model supports 15 languages. Note that the XLM leverages the alignment information from languages through TLM task. In addition, it can test the recognition rate to an unseen language identity since XLM is trained with 15 languages.

*XLM-Roberta* (Conneau et al., 2020) is a multilingual language model based on Roberta (Liu et al., 2019). It is trained on CommonCrawl corpora with 12 layers and 768 hidden states. The vocabulary size is 250k, The tool for tokenization is free, namely, the Sentence Piece (Kudo & Richardson, 2018). The only task is the dynamic mask language model. XLM-Roberta supports 100 languages.

### Model architecture

**Figure 1:**
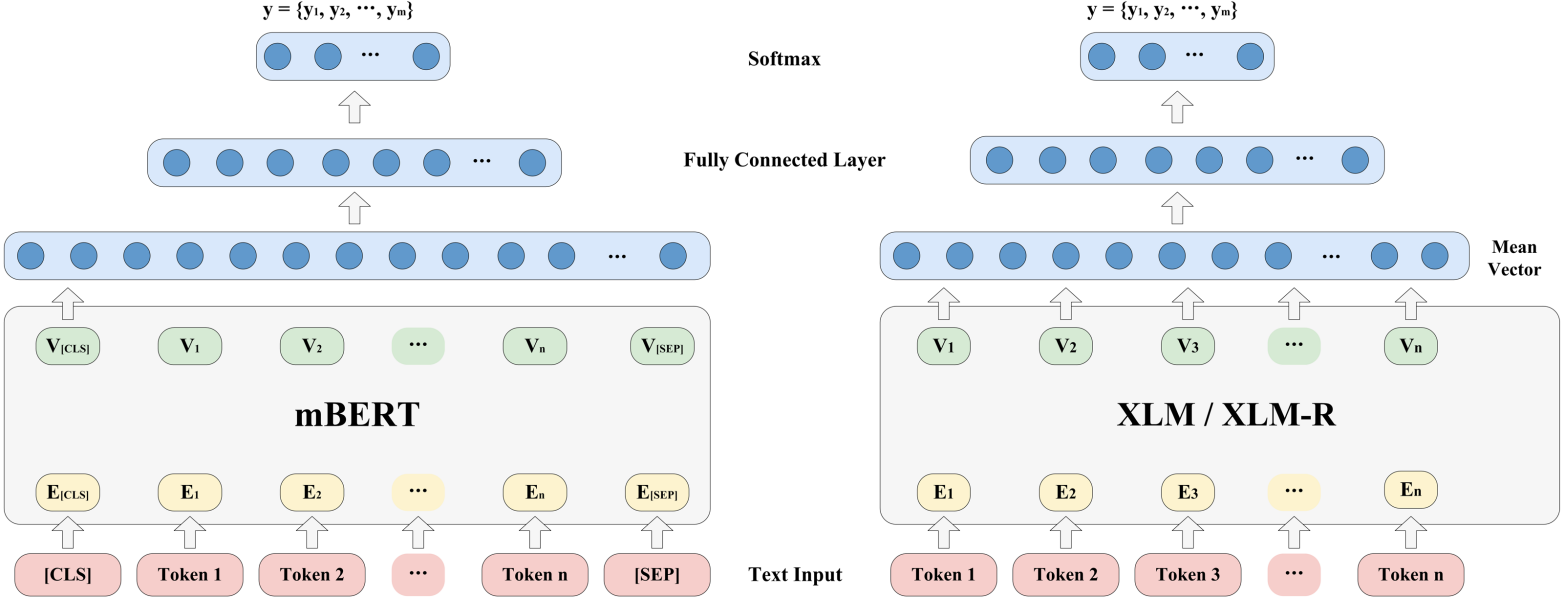
Model architecture for probing language identity.

Figure 1 shows the model architecture for probing the ability of the pre-trained multilingual models to encode language identities. Given a sentence and all classes of a certain typological feature {y_k_ —s}= {y_k_ —t_1_, t_2_, …, t_n_ }, *k* = 1,2, …,m. y_k_ represents a class of a certain typological feature, and s = {t1, t2, …, tn} represents a sentence, where t_i_(1 ≤i ≤ n) refers to i^th^ token in the sentence. During training, if the pre-trained multilingual encoder is mBERT, we will add two additional tokens, [CLS] and [SEP], at the beginning and end of the sentence. The [CLS] token can be used for classification for this sentence later. We will take [CLS] embedding as the output of mBERT; if the pre-trained multilingual encoder is XLM or XLM-R, the basic principle is the same above. The main difference is that we will average these token embeddings to obtain a mean vector, the formula is as follows:

(1) \begin{eqnarray*}{\mathrm{V }}_{\text{mean}}= \frac{1}{\mathrm{n}} \sum _{\mathrm{i}=1}^{\mathrm{n}}{\mathrm{V }}_{\mathrm{ i}}\end{eqnarray*}

where V_i_ represents the i^th^ output embedding, and n is the number of tokens in a sentence.

After obtaining the output vector of the encoder, we will input it into a fully connected layer to obtain the score vector about typological class, y ∈ R^m×1^, where m is the number of class of a certain typological feature. Furthermore, we use the softmax function to normalize y to obtain the conditional probability distribution P(y) ={y_1_, y_2_, …, y_m_}. The formulas are as follows:

(2) \begin{eqnarray*}\mathrm{y} & ={\mathrm{W}}_{1}{\mathrm{V }}_{[CLS]}+{b}_{1}\end{eqnarray*}

(3) \begin{eqnarray*}\mathrm{y} & ={\mathrm{W}}_{1}{\mathrm{V }}_{mean}+{b}_{1}\end{eqnarray*}

(4) \begin{eqnarray*}\mathrm{P}({\mathrm{y}}_{\mathrm{i}})& = \frac{exp({\mathrm{y}}_{\mathrm{i}})}{\sum _{\mathrm{j}=1}^{\mathrm{m}}exp({\mathrm{y}}_{\mathrm{j}})} \end{eqnarray*}

where W_1_ is the weight matrix, b_1_ is the bias vector. The cross-entropy loss function is used to train and update the parameters of the model through a backpropagation algorithm, the formula is as follows:

(5) \begin{eqnarray*}\text{loss} =-\sum _{\mathrm{s}\in S}\sum _{\mathrm{i}=1}^{m}{P}_{\mathrm{ i}}^{\mathrm{t}}(\mathrm{s}){\log \nolimits }_{2}({P}_{\mathrm{i}}^{\mathrm{p}}(\mathrm{s}))\end{eqnarray*}

where S is the training set, s is a certain sentence in the training set. ${P}_{\mathrm{i}}^{\mathrm{t}}(\mathrm{s})$ is the ground truth probability distribution of the typological class of s, and ${P}_{\mathrm{i}}^{\mathrm{p}}(\mathrm{s})$ is the predicted probability distribution of the typological class of s.

During testing, we will compute the prediction accuracy of each encoders on each typological feature. And then compute the arithmetic average among all features, which is an indicator of encoding identity information of the language.

(6) \begin{eqnarray*}\mathrm{Acc} = \frac{1}{\mathrm{M}} \sum _{\mathrm{k}=1}^{\mathrm{M}}{\mathrm{P}}_{\mathrm{ k}}\end{eqnarray*}

where P_k_ is the prediction accuracy on a certain feature. M represents the number of features.

We keep the parameters of the pre-trained multilingual encoder fixed in the experiment. To make the model focus as much as possible on the information provided by the multilingual encoder, the number of hidden neurons in fully connected layer is 100. In addition, we adopted “leave-one-out cross validation” in our experiment. For each typological feature prediction experiment, all sentences from 35 languages are used to train the model to learn this typological feature, and the remaining language is used for prediction (for XLM, we only use the 10 languages supported by the encoder). Considering both the equipment capacity and the processing speed, we set the training epoch as 5, the batch size as 256, and the dropout as 0.5.

Other parameter settings were attempted, but our key findings did not change.

### Experiment

### Typological features

In our experiment, the typological features are all from two databases: WALS and SSWL:

The World Atlas of Language Structures, WALS (https://wals.info/), is a large database of typological features (phonological, grammatical, lexical) of 2662 languages (Dryer & Haspelmath, 2013). In WALS, each feature means a structural property of language. For example, for the feature “138A: Tea”, the typologist annotates this feature as “Words derived from Sinitic ‘cha”’, “Words derived from Min Nan Chinese ‘te”’ or “Others”. Considering the annotation sparsity and differentiation of each feature across languages, we selected a total of 55 features from the areas of lexicon, word order, and syntax, *etc.* The syntactic features in WALS all belong to nominal syntax. To cover more typological features, we screened out a small portion of features from SSWL.

Syntactic Structures of the World’s Languages, SSWL (http://terraling.com/) is a publicly accessible and open-ended database for language research. This database stores morphological, syntactic, and semantic patterns of 319 languages. We selected 40 typological features from SSWL. These features cover many areas, such as word order, syntax and noun. In SSWL, each feature is labeled with ”yes/no”. To be consistent with the way WALS is annotated, we merged some features of SSWL and used the sub-features originally labeled as “yes” as the new label. For example, ”O 02_1:Def Mass Can be bare”, ”O 02_2:Def Mass Can have an article” and ”O 02_2:Def Mass Must have an article” are merged into ”O 02: Definite Mass”. By this way, 40 features were merged into 12 features.

Table 1 shows the 67 features collected from the WALS and SSWL databases, along with their Id and areas. Depending on the areas, 67 features can be categorized into Nominal Category, Simple Clauses, Verbal Category, Word Order, Lexicon, Morphology and Syntax.

### Dataset

We finally selected 36 languages considering the annotation coverage in WALS and SSWL as well as the languages supported by multilingual encoders. There are 128 language families and hundreds of language groups in the world (Campbell, 2008). The languages in our experiment involves nine languages families, such as Indo-European, Altaic and Uralic language family, and 20 language groups, such as Germanic, Roman and Slavic language groups. Table 2 shows the 36 selected languages and the corresponding language groups, where each language will be represented in terms of ISO 639-1 code for brevity.

**Table 1:** Typological features.

**Area**	**Id**	**Feature name**	**Area**	**Id**	**Feature name**
Word Order	87A	Order of Adjective and Noun	Nominal Categories	33A	Coding of Nominal Plurality
	88A	Order of Demonstrative and Noun		53A	Ordinal Numerals
	143A	Order of Negative Morpheme and Verb		51A	Position of Case Affixes
	83A	Order of Object and Verb		37A	Definite Articles
	82A	Order of Subject and Verb		38A	Indefinite Articles
	81A	Order of Subject, Object and Verb		47A	Intensifiers and Reflexive Pronouns
	144A	Position of Negative Word With Respect to Subject, Object, and Verb		45A	Politeness Distinctions in Pronouns
	143F	Postverbal Negative Morphemes		50A	Asymmetrical Case-Marking
	143E	Preverbal Negative Morphemes		49A	Number of Cases
	97A	Relationship between the Order of Object and Verb and the Order of Adjective and Noun		46A	Indefinite Pronouns
	85A	Order of Adposition and Noun Phrase		36A	The Associative Plural
	86A	Order of Genitive and Noun		52A	Comitatives and Instrumentals
	95A	Relationship between the Order of Object and Verb and the Order of Adposition and Noun Phrase		57A	Position of Pronominal Possessive Affixes
	90A	Order of Relative Clause and Noun		O01	indefinite mass
	96A	Relationship between the Order of Object and Verb and the Order of Relative Clause and Noun		O02	definite mass
	92A	Position of Polar Question Particles		O04	definite singular
	94A	Order of Adverbial Subordinator and Clause		O06	definite plural
	93A	Position of Interrogative Phrases in Content Questions		Verbal Categories	69A	Position of Tense-Aspect Affixes
	91A	Order of Degree Word and Adjective			70A	The Morphological Imperative
	90C	Postnominal relative clauses			72A	Imperative-Hortative Systems
	21&22	Order of Pronominal Possessor and Noun			71A	The Prohibitive
	C01	Complementizer Clause	75A		Epistemic Possibility
	N3_01	Order of Noun, Adjective and Demonstrative	76A		Overlap between Situational and Epistemic Modal Marking
	N3_07	Order of Noun, Numeral and Demonstrative	74A		Situational Possibility
	Simple Clauses	112A	Negative Morphemes		78A	Coding of Evidentiality
		116A	Polar Questions		77A	Semantic Distinctions of Evidentiality
		101A	Expression of Pronominal Subjects		73A	The Optative
		119A	Nominal and Locational Predication		Nominal Syntax	63A	Noun Phrase Conjunction
		118A	Predicative Adjectives	N2		A noun phase containing Num (N Num or Num N) in a definite context
		120A	Zero Copula for Predicate Nominals	Syntax	Q06	Polar question
115A		Negative Indefinite Pronouns and Predicate Negation	Q09		Affirmative answer
117A		Predicative Possession	Q16		NEGA_Negative answer
Lexicon	138A	Tea	Morphology	26A	Prefixing vs. Suffixing in Inflectional Morphology
	129A	Hand and Arm			

**Notes.**

The “id” tag is from WALS and SSWL.

**Table 2:** Sample languages.

**Language**	**Group**		**Language**	**Group**		**Language**	**Group**
Danish(da)	Germanic		Bulgarian(bg)	Slavic		Japanese(ja)	Isolated
Dutch(nl)	Germanic		Czech(cs)	Slavic		Korean(ko)	Altaic
English(en)	Germanic		Polish(pl)	Slavic		Turkish(tr)	Turkic
Icelandic(is)	Germanic		Russian(ru)	Slavic		Hindi(hi)	Indic
Norwegian(no)	Germanic		Serbian(sr)	Slavic		Nepali(ne)	Indic
Swedish(sv)	Germanic		Ukrainian(uk)	Slavic		Estonian(et)	Finnic
Catalan(ca)	Romance		Albanian(sq)	Albanian		Hungarian(hu)	Ugric
French(fr)	Romance		Basque(eu)	Basque		Chinese(zh)	Sign Languages
Italian(it)	Romance		Indonesian(id)	Malayo-Sumbawan		Greek(el)	Greek
Portuguese(pt)	Romance		Kannada(kn)	Southern Dravidian		Hebrew(he)	Semitic
Romanian(ro)	Romance		Lithuanian(lt)	Baltic		Persian(fa)	Iranian
Spanish(es)	Romance		Irish(ga)	Celtic		Vietnamese(vi)	Viet-Muong

For each language, we extracted 10,000 sentences from the News section of the Leipzig multilingual corpus (https://wortschatz.uni-leipzig.de/en/download). Since Leipzig is a multilingual corpus formed by aggregating different monolingual corpora, there are no semantic relations between sentences from different languages.

### Experimental results

Next, we introduce experimental results from the language level and the typology level respectively.

### Language level

The models predict each typological feature of this language, and then we compute the average as the preservation degree of language identity. To compare the abilities of pre-trained multilingual models to encode language identities, we used ”Random BERT” as the baseline, drawing from the idea of Tenney et al. (2018) using the same architecture as the BERT, yet Random BERT randomizes all weights of each layer above the lexical layer (layer 0). The results are shown in Table 3.

**Table 3:** Results of encoding language identities from each pre-trained multilingual model.

**Lang**	**mBERT**	**XLM-R**	**XLM**	**Baseline**	**Lang**	**mBERT**	**XLM-R**	**XLM**	**Baseline**
bg	72.42%	74.07%	71.87%	56.66%	hu	57.42%	59.82%	N/A	43.09%
zh	59.37%	57.24%	54.85%	27.16%	is	69.31%	73.53%	N/A	71.25%
en	82.45%	80.01%	74.91%	49.57%	id	50.41%	49.66%	N/A	33.07%
fr	79.87%	78.19%	74.48%	52.67%	ga	60.21%	60.44%	N/A	53.06%
el	75.81%	75.53%	71.46%	62.00%	it	82.69%	82.39%	N/A	65.30%
hi	59.46%	68.60%	58.34%	42.54%	ja	52.27%	45.14%	N/A	27.29%
ru	73.45%	75.12%	66.07%	57.23%	kn	63.80%	60.00%	N/A	43.27%
es	89.37%	87.19%	75.78%	43.12%	ko	66.45%	70.71%	N/A	31.03%
tr	57.43%	49.12%	43.34%	34.73%	lt	73.19%	65.49%	N/A	47.18%
vi	67.32%	55.19%	48.30%	42.43%	ne	67.25%	63.74%	N/A	38.37%
sq	68.92%	64.37%	N/A	54.40%	no	93.45%	89.07%	N/A	65.05%
eu	49.99%	46.72%	N/A	28.04%	fa	56.07%	51.28%	N/A	32.27%
ca	82.26%	77.67%	N/A	30.12%	pl	74.78%	73.63%	N/A	65.05%
cs	69.23%	66.02%	N/A	43.80%	pt	81.94%	83.74%	N/A	50.67%
da	**95.56%**	**91.81%**	N/A	64.76%	ro	75.63%	73.26%	N/A	52.91%
nl	73.12%	72.10%	N/A	43.42%	sr	79.81%	83.12%	N/A	51.49%
et	72.07%	72.77%	N/A	55.32%	sv	89.14%	81.64%	N/A	48.47%
he	58.17%	47.07%	N/A	42.64%	uk	89.88%	91.61%	N/A	35.61%
					**Ave**	71.39%	69.36%	63.94%	46.81%

**Notes.**

“N/A” means XLM model do not support this language. The bolded numbers are the maximum values for each model among all sample languages; the underlied numbers are the minimum values for the models among all sample languages.

(1) Comparison of languages

Based on the results in Table 3, we found that the identity of the same language was consistently preserved in the three models. Among all the 10 languages supported by XLM, the three models reserve the language identity for Spanish best, while Turkish is the worst. Among all the sample languages, mBERT and XLM-R encoded the language identity of Danish best. Danish belongs to Germanic language group, the Indo-European language family (the main language family). Verbs in Danish possess different forms according to different tenses, but there is no variation in person and number, indicating that the change of word form is simple and conforms to the grammatical norms. Out of the 67 typological features in Table 1, Danish has annotated results for 55 features. And 44 of them are results same as the most labels on each feature. The degree of grammatical standardization is 80%. From the perspective of typology, it is shown that mBERT and XLM-R do not require much training and memory when encoding the language’s identity, and the preserving effect of Danish is relatively high. In contrast, mBERT and XLM-R perform poorly in Japanese. So far, the language family of Japanese is unknown yet. Although there are many hypotheses, no unified view has been reached (Kindaichi, 2017). Japanese is a subject-object-predicate structured and cohesive language, and its writing system is more complicated than other languages. Among all the 67 typological features, Japanese has annotated results on 66 features, 19 of which are results same as the most label on each feature. The degree of grammatical standardization is poor. This indicates that both models need to store additional information about typological properties of the language, so the preserving effect is very low.

**Figure 2:**
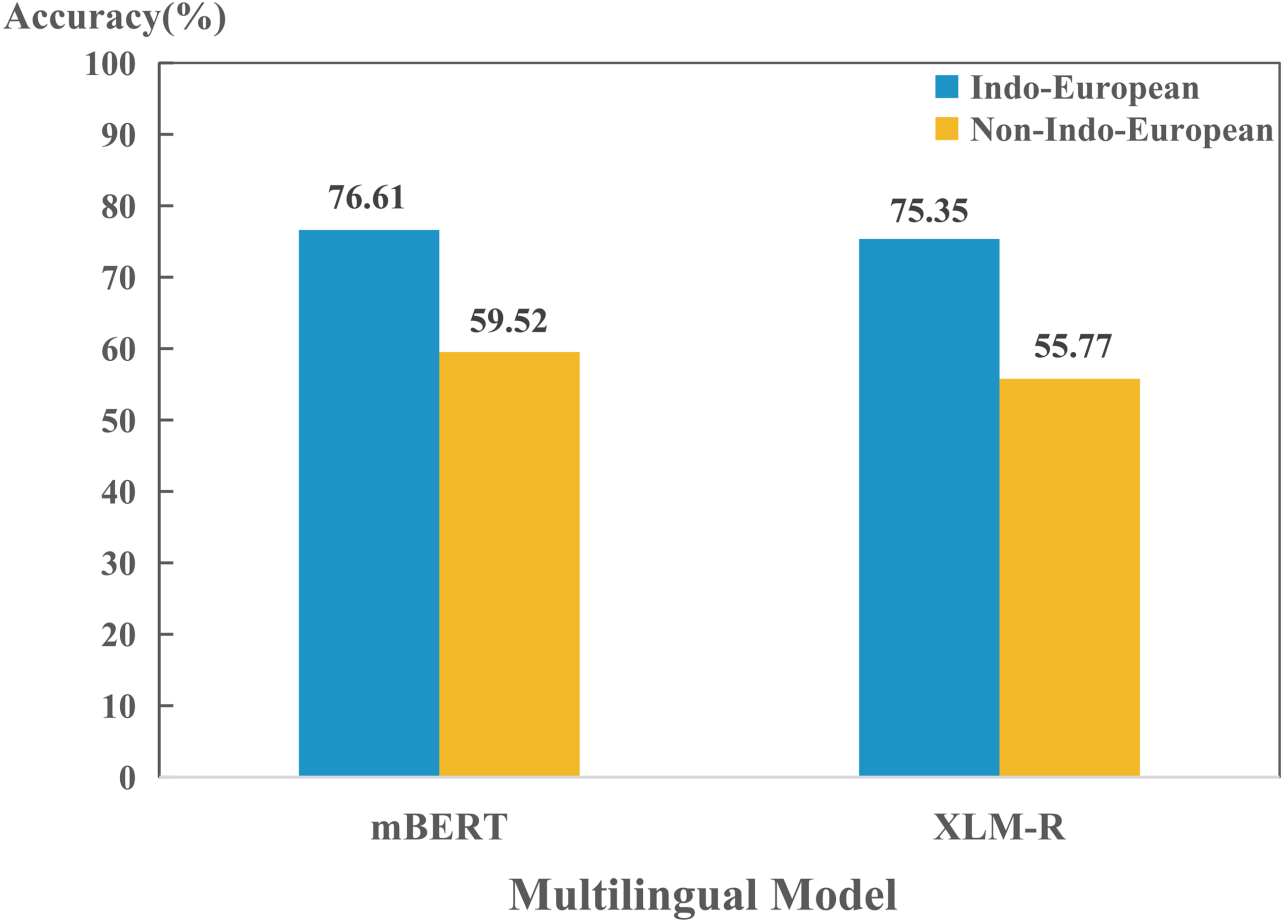
Performance for each model on encoding language identities of (non-)Indo-European language family.

**Figure 3:**
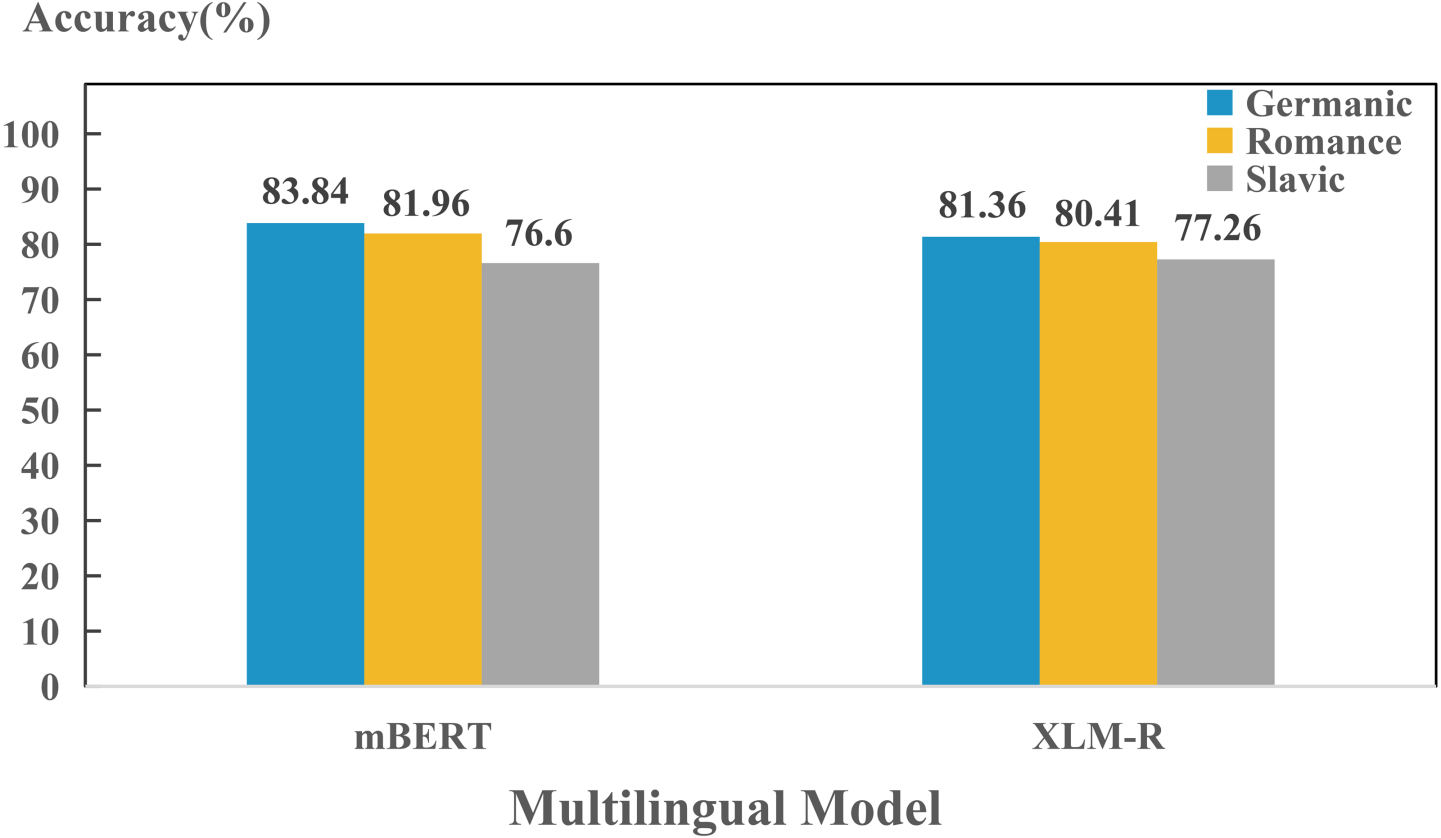
Performance for each model on encoding language identities of three language groups.

**Figure 4:**
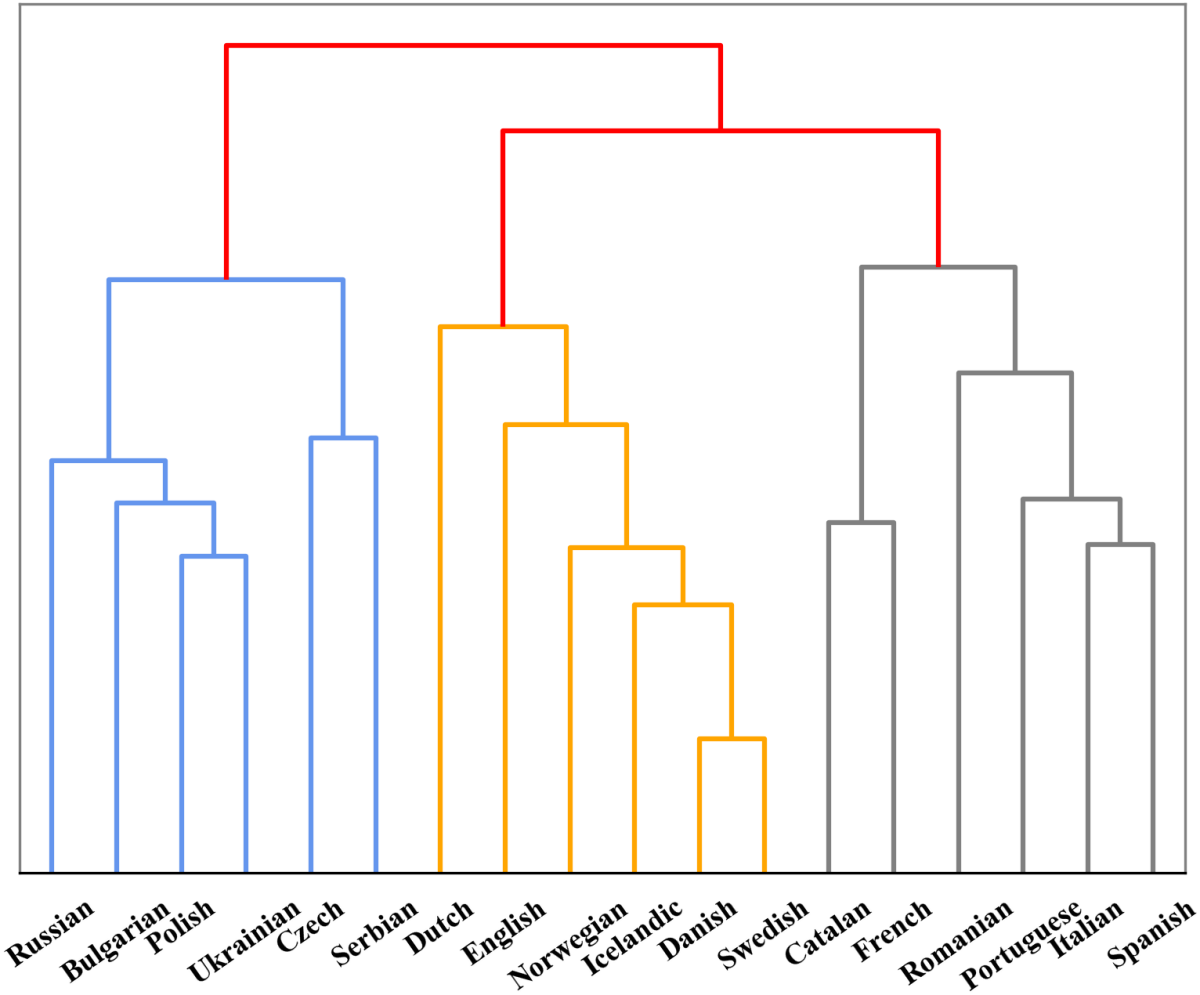
Clustering tree of three language groups based on typological results from XLM-R.

In addition, we also found that models perform differently in encoding different language. To further investigate the reason behind this phenomenon, we compared the performance between Indo-European and non-Indo-European languages. As shown in Fig. 2, there is significant differences in encoding language identities of both language families for mBERT and XLM-R. This might be due to the fact that most of the 100+ languages collected are Indo-European language, so the two models can learn the properties of the language family well. Furthermore, since we collected many languages from Germanic, Roman and Slavic language groups in the Indo-European language family, we also studied the encoding abilities of mBERT and XLM-R in terms of language groups, as shown in Fig. 3. The overall performance of both models on each group is high, since the three language groups all belong to the Indo-European language family, and the property differences within each language group are not significant. Compared with Germanic and Roman language groups, Slavic language group possesses more free syntax structure (Sussex & Cubberley, 2006). This may affect the encoding language identities of the language group by mBERT and XLM-R. Therefore, these two models perform slightly worse in this language group. To further show the accuracy of the models in encoding language identities, we used the results of typological features predicted by XLM-R (missing values were filled with the mean value) to perform hierarchical clustering for languages in the three language groups. In this experiment, we used the correlation distance method to calculate the farthest neighbor for clustering. The clustering results are shown in Fig. 4. It shows that the languages in each language group are well clustered according to the results of XLM-R.

(2) Comparison of models in encoding language identity

Based on the results in Table 3, the abilities of encoding language identities of these models are in order: mBERT > XLM-R > XLM >> random BERT. We observed that the performance of mBERT is better than XLM-R, it might be because that the Next Sentence Prediction (NSP) task plays an effective role in preserving language identity when training the BERT model. BERT requires determining whether two sentences are from the same language and whether they are adjacent to each other. Among the 10 languages supported by XLM, XLM is less capable of encoding language identity than mBERT and XLM-R. Unlike the models trained on different monolingual corpus, XLM takes XNLI as the training task during pre-training, and the corpus for training is bilingual aligned sentences. Therefore, XLM has learned some alignment information between languages during pre-training. Although this training method allows XLM to perform better on cross-lingual transferring tasks, it might also weaken the potential ability of XLM to preserve language identities; Random BERT performs the worst. This also reflects the fact that pre-trained multilingual models can potentially encode language identities. However, the preserving effect of Random BERT is not very poor. This may be because the structure of BERT achieves general results on many natural processing tasks, even if the parameters are randomly initialized (Zhang & Bowman, 2018).

### Typology level

To indicate the extent of typological features captured by each model, we calculate the average of all languages predicted in terms of typological features.

(1) Comparison of typological areas

**Figure 5:**
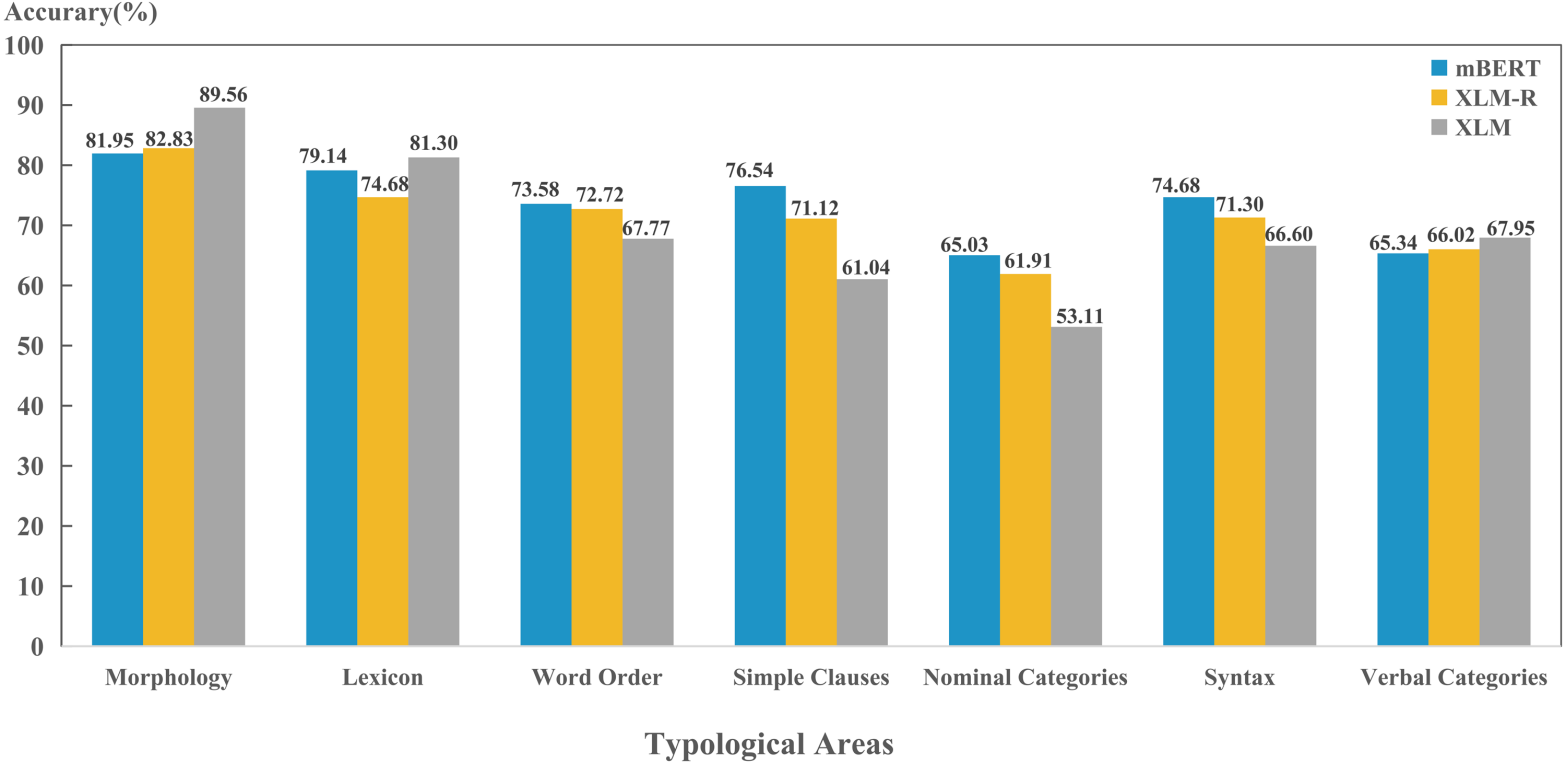
Performance on per typological area for each model.

The results in Fig. 5 shows that three models were able to capture four typological areas well, including morphology, lexicon, word order and syntax; while perform poorly on the features in nominal category and verbal category. This suggests that these four typological areas can reflect the properties of the language well and play a vital role for the models in encoding language identity. Although models perform best in the morphology area, our experiment only adopted one feature in morphology (“26A”), which has limited representativeness. In the future, we will consider more morphological features and obtain more objective and scientific results. In addition, these four areas of features are all superficial and formalized features, so that three models can learn and capture better during pre-training. Both nominal category and verbal category belong to the grammatical category. The grammatical category is the generalization of the grammatical meaning expressed by various grammatical forms. In accordance with grammatical form, it includes all explicit grammar and implicit grammars; In accordance with grammatical sense, it includes all structural meanings, functional meanings and descriptive meanings (Bybee, 1998). This suggests that these two typological areas cover semantic information in depth, which the model fails to encode.

(2) Comparison of models in encoding typological features

**Figure 6:**
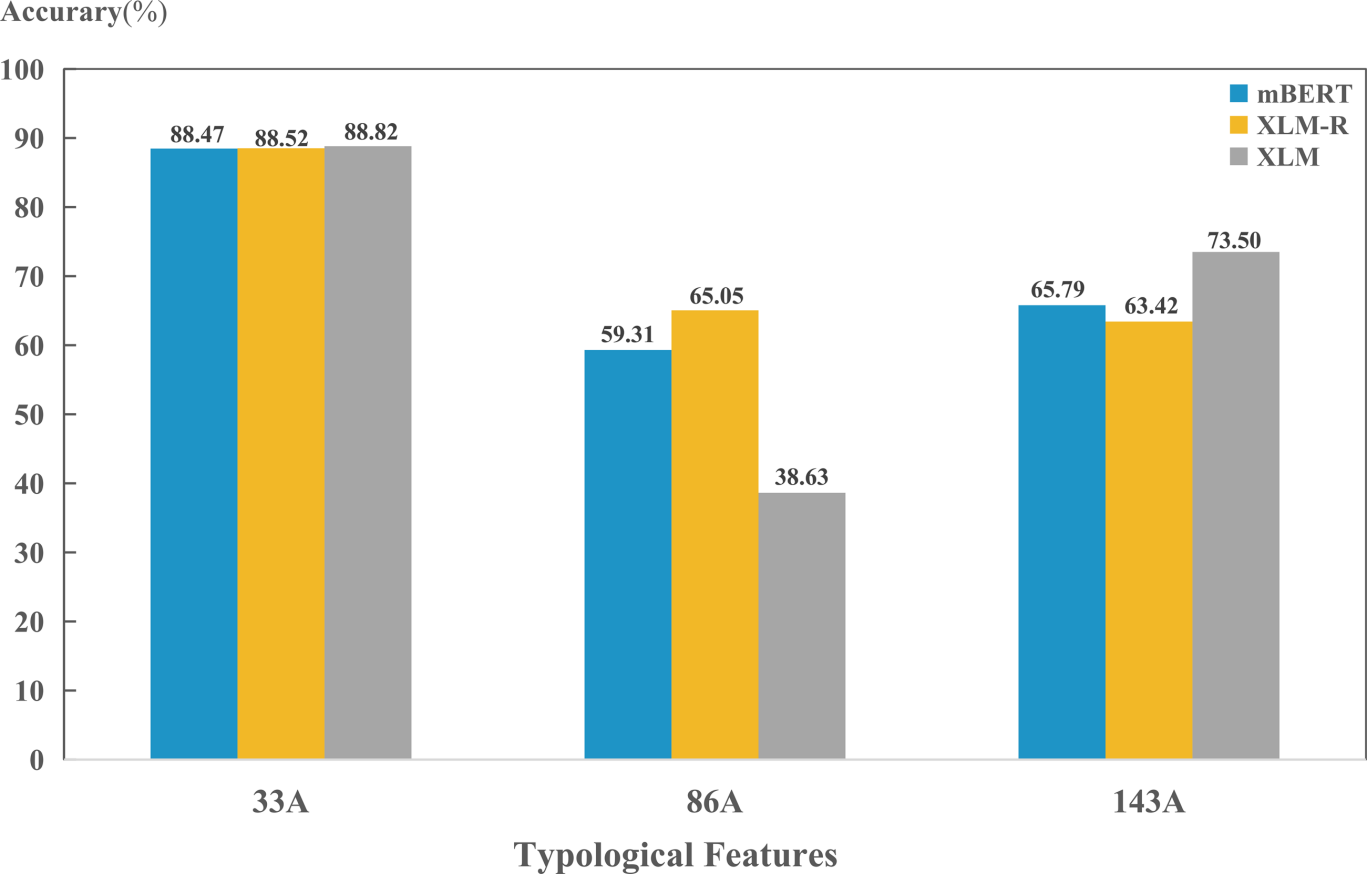
Performance on three example typological features.

We also found that the predictions of the three models on the same feature were generally consistent; while there were some features that differed significantly among the three models. As shown in Fig. 6, the three models performed similarly on the feature “33A”. However, XLM is significantly lower than the other two on feature “86A”, and significantly higher than the other two on feature “143A”.

**Figure 7:**
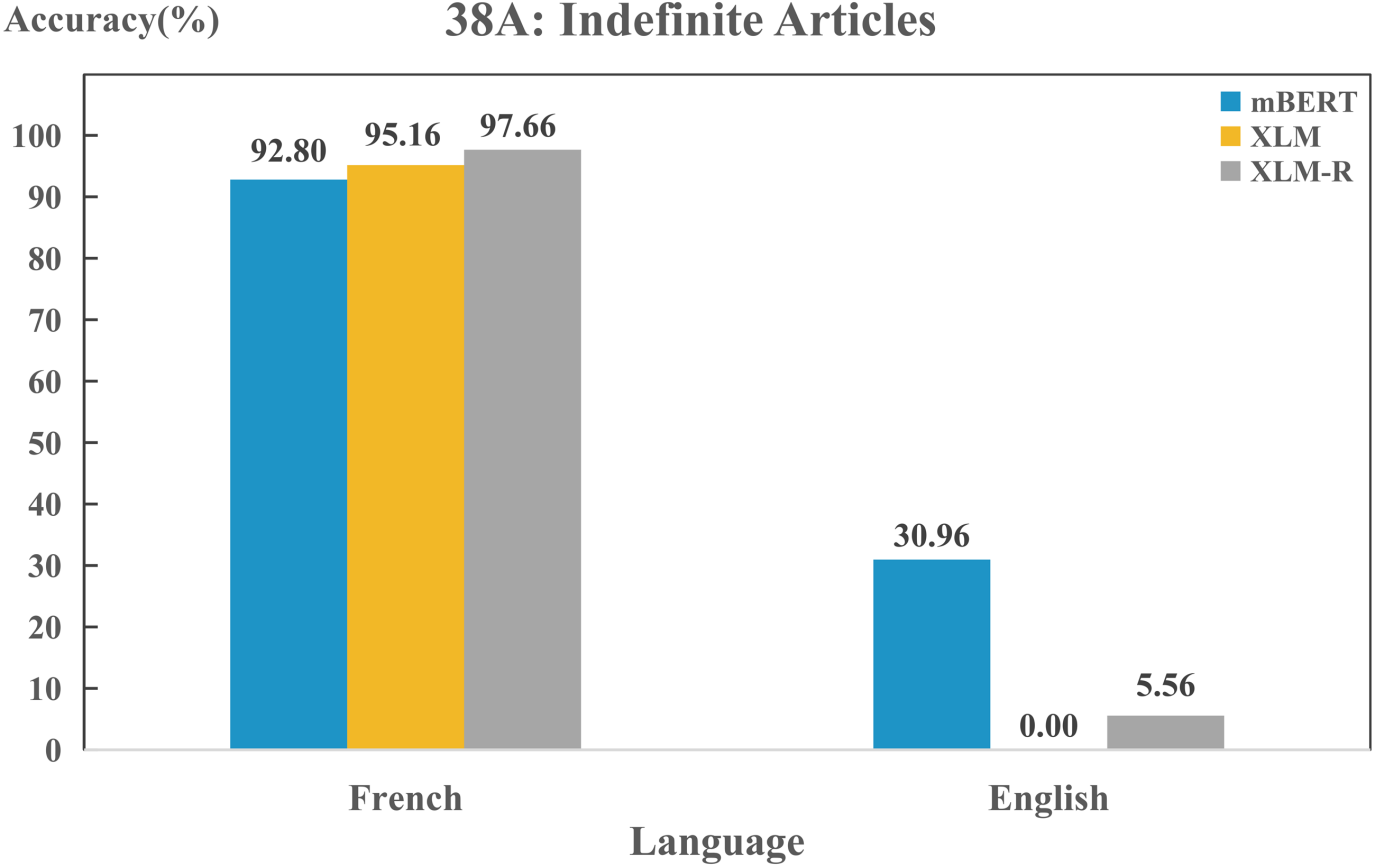
Performance on feature “38A” of English and French for each model.

In addition, we found some differences between the models when we delved into the features of a particular language. Three models could perform differently on the same feature in any two languages, even they might possess different capacities in capturing the same feature of the same language. As an example, in Fig. 7, there is a clear difference in the use of “indefinite articles” between English and French. In WALS, English is labeled as “Indefinite word distinct from ‘one”’, while French as “Indefinite word same as ‘one”’. In fact, this is consistent with the actual situation. The indefinite article is represented by “a/an” in English, which is different from the expression of the numeral “one”. In French, the numeral and the indefinite article are both represented by “un/une”. All three models show a high performance on this feature in French, while they perform poorly in English. Such results also disclose that typological features can describe the properties of language and reflect the language identity well. Furthermore, Fig. 7 also shows that the three models exhibit significant difference on feature “38A” in English, the predicting effect of mBERT (30.96%) is significantly higher than the other two (0% and 5.56%).

### Probing Across Layers

### Language level

(1) Comparison of languages across layers

**Figure 8:**
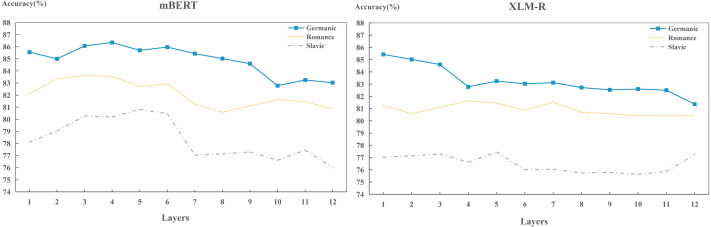
Layer-wise performance for each model on per language group.

In the cross-layer experiments, we found that the capacities of different models to encode different language identity across layers are distinct. To further investigate the capacities across layers of each model to encoding different language identities, we carried out layer-wise detection experiments on Germanic, Roman and Slavic language groups, as shown in Fig. 8. We found that in terms of performance, the order of the language identities preserved by two models are: Germanic > Romance > Slavic, which is consistent with the findings in Fig. 3. This indicates that during training process, each layer has already learned the ability to preserve different languages.

(2) Comparison of models in encoding language identity across layers

**Figure 9:**
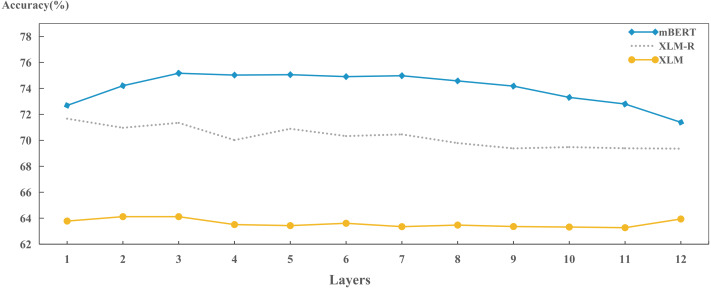
Layer-wise performance for each model on encoding language identities.

We also explored the capabilities of different models in preserving language identity across layers, as shown in Fig. 9. We found that the ability ranking in the same layer is: mBERT > XLM-R > XLM, which is consistent with the results in Table 3. It also shows that each hidden layer has already started to learn how to encode language identity during training. Differences in ability between the models have emerged for each hidden layer.

In addition, the results in Fig. 9 also show that each model has some cross-layer variation in encoding language identities. That is, the XLM remains stable, the XLM-R fluctuates slightly, and the mBERT varies dramatically. As for the performance of XLM, it shows that during model training, bilingual alignment plays an effective role in each layer. For the XLM-R, the performance gradually decreases as the increase of layer. This may be because the dynamically masked language object during training can help the model encode the language identity in lower layers; mBERT performs best in middle layers (layers 3 to 8), and its accuracy gradually decreases after layer 8. This might also be because mBERT uses different monolingual texts as training data and needs to encode language features in the model so that it can perform well in MLM and NSP tasks. Because higher layers of mBERT will be fine-tuned and applied into downstream tasks, the top layers will weaken the identity information of the language to some extent.

### Typology level

**Figure 10:**
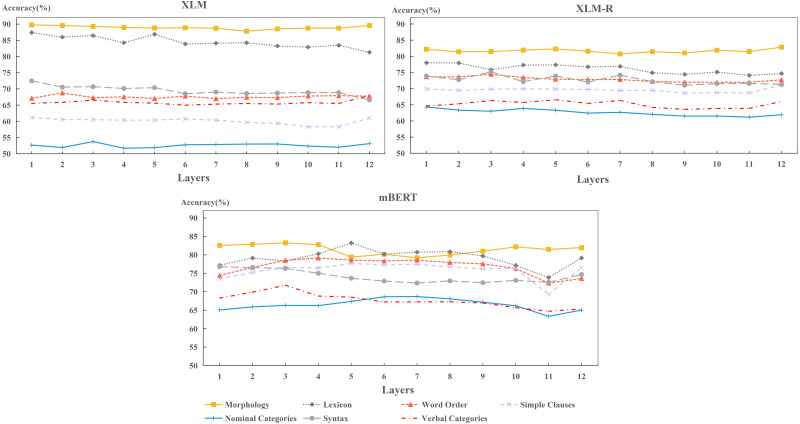
Layer-wise performance for each model on per typological area.

In this section, we explore the capability of each model to encode typological features across layers. The results are shown in Fig. 10.

(1) Comparison of typological features across layers

Figure 10 shows that the hidden layers of each model perform best on morphology and lexicon, while worst on nominal categories and verbal categories. This result is consistent with the findings in Fig. 5 as shown above. It indicates that the performance of each hidden layer determines the performance of final layer. In fact, some easy-to-learn features can be encoded well in the hidden layers; while those hard-to-learn features are not well preserved in these layers during training.

(2) Comparison of models in encoding typological features across layers

From Fig. 10, we observed that the performance of XLM-R and XLM keep stable in encoding each typological area, while mBERT fluctuates severely. The performance of XLM-R and XLM indicates that each hidden layer has consistently acquired the competence in encoding typological features. As for mBERT, it shows that each layer has not exactly the same ability in encoding various features. Specifically, mBERT can capture syntactic information in the lower layers (layers 1 to 3), while encoding word-level information in the middle layers (layers 3 to 9), such as lexicon and word order. This finding is consistent with the previous work (Jawahar, Sagot & Seddah, 2019). It also shows that mBERT learns how to organize words into sentences in the lower layers and memorizes the vocabulary information of the language in middle layers.

### Case study

In this section, we look specifically at the ability of these models to preserve the language identity of Chinese. Chinese is the most spoken language in the world and is widely used in the mainland China and Chinese communities in Singapore, Malaysia, the United States, Canada, and Australia. Chinese belongs to the Sino-Tibetan language family and is a branch of analytical languages. The writing system is the Chinese character, which is a kind of logogram and have both ideographic and phonetic functions. From the perspective of language typology, Chinese as a whole is an analytic language, but there are still some inflections, adhesions and even polysynthetic phenomena. This language is a widely used and extremely special. Therefore, we have analyzed Chinese in detail. Table 4 shows the results of Chinese on various typological features from mBERT. The underlined numbers are the accuracy values at a pretty low level. By looking up the annotation results on these typological features, we found that the annotation results of Chinese are extremely different from other languages. For example, as to feature “85A”, it means “Order of Adposition and Noun Phrase”. The label of Chinese is “No dominant order”, while others are often either “Postpositions” or “Prepositions”. Such situation makes it difficult for the model to make accurate predictions, so the accuracy values on these features are pretty low.

**Table 4:** Results of the mBERT model on Chinese typological features.

**Id**	**Acc(%)**	**Id**	**Acc(%)**	**Id**	**Acc(%)**	**Id**	**Acc(%)**
87A	74.44%	93A	87.12%	33A	99.98%	63A	36.52%
88A	99.21%	91A	99.96%	53A	41.08%	N2	23.96%
143A	85.16%	90C	N/A	51A	85.02%	Q06	N/A
83A	63.86%	21&22	93.96%	37A	N/A	Q09	0.77%
82A	**100.00%**	C01	N/A	38A	N/A	Q16	92.81%
81A	79.98%	N3_01	16.70%	47A	99.79%	112A	78.54%
144A	63.13%	N3_07	25.60%	45A	6.23%	116A	99.77%
143F	95.90%	69A	99.58%	50A	33.64%	101A	64.91%
143E	93.15%	70A	11.76%	49A	76.22%	119A	59.35%
97A	13.74%	72A	98.77%	46A	0.06%	118A	35.32%
85A	0.00%	71A	84.61%	36A	11.42%	120A	97.72%
86A	90.19%	75A	56.93%	52A	19.12%	115A	99.73%
95A	0.02%	76A	11.30%	57A	99.91%	117A	28.09%
90A	7.37%	74A	92.03%	O01	0.28%	138A	96.49%
96A	0.00%	78A	33.77%	O02	97.00%	129A	72.08%
92A	52.66%	77A	54.47%	O04	6.01%	26A	99.56%
94A	N/A	73A	99.98%	O06	74.97%	**All**	**59.37%**

**Notes.**

“N/A” means the typological feature represented by the id are not annotated in Chinese. The bolded numbers are the maximum values for mBERT among all typological features; the underlied numbers are the minimum values for mBERT among all typological features.

**Figure 11:**
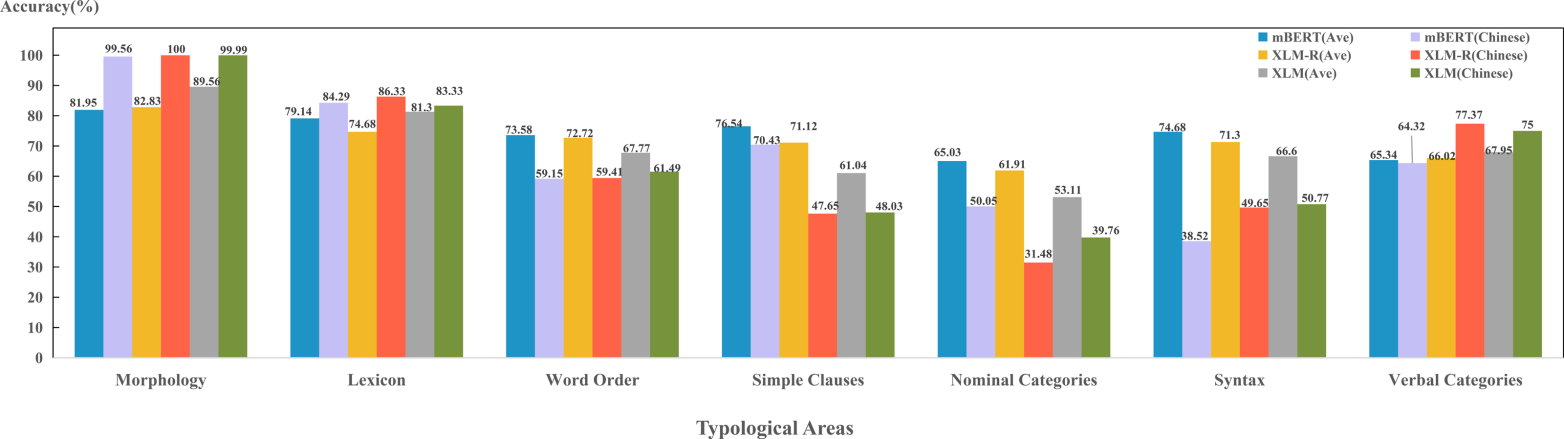
Performance of each model in the field of Chinese typology.

Figure 11 shows the models abilities to predict various typological properties of Chinese. The abilities of three models to capture Chinese morphology and lexical features are higher than the average of sample languages. This is due to the lack of inflection in Chinese expressions. For example, the genitive case of Chinese, where a Chinese character “的” is usually attached to personal pronouns, such as “你”, “我”, “他” *etc.* Indo-European languages, such as English, on the other hand, will use different words, such as “my”, “your”, “her”, *etc.* This feature of Chinese also facilitates the model to capture such morphological and lexical features. However, for features belonging to categories of word order, syntax and grammar, the models’ abilities in Chinese are far below the average of sample languages. This is because Chinese is a kind of “paratactic” language. As long as the meaning is correct, it is not so necessary to consider the order of language components, such as the example sentences, “饭，我吃了。” (meal, I ate), “我吃了饭” (I ate the meal) and “吃饭了，我” (ate the meal, I). All these three cases represent the same meaning. This makes the word order and syntax of Chinese delicate and complicated. In addition, there are a large number of Chinese language users, and the convenience of the Internet enables them to innovate expressions. This could further promote the flexibility of Chinese expressions, and thus it is difficult for the models to accurately encode these features.

**Figure 12:**
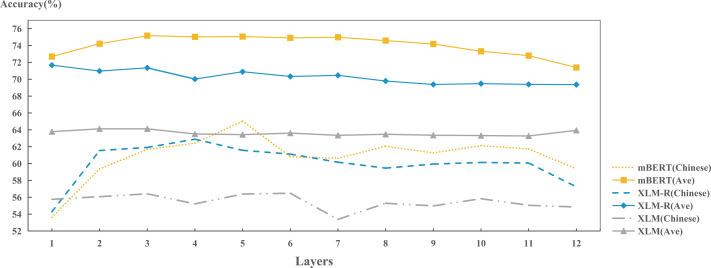
Layer-wise performance for each model on encoding Chinese language identity.

In addition, we also investigated the ability of each model to preserve Chinese language features in each layer, as shown in Fig. 12. For each layer, the ability of each model to preserve language identity of Chinese is generally lower than the average of sample languages. However, it still reflects that: mBERT > XLM-R > XLM. From the across-layer perspective, the fluctuation of XLM across layers have not changed much. There is a slight decrease in performance at layer 7. While the performance on XLM-R and mBERT fluctuates more significantly. At the lower layers (layers 1 to 4), the performances of both models increase with the increase of layer. In contrast, the capacity of the intermediate layers tends to stabilize. Later, the performances drop slightly at layer 11 and 12. In the lower layer (layers 1 to 4), XLM-R performs slightly better than mBERT. While in the middle layer, it is surpassed by mBERT. We try to explain this phenomenon. XLM-R may contain more general information in lower layers (Li et al., 2020); While BERT can capture surface features in lower layers, syntactic features in middle layers and semantic features in higher layers (Jawahar, Sagot & Seddah, 2019). Because the surface features of Chinese are not very obvious to be recognized, when mBERT learns the shallow features of Chinese at the lower level, its recognition ability on Chinese is lower than XLM-R’s. However, when mBERT starts to learn syntactic information in middle layer, the situation changes, so it can surpass XLM-R.

## Conclusions

In this paper, we explored the abilities of pre-trained multilingual models to encode language identity. We found that mBERT and XLM-R have better ability to preserve language identity compared to XLM. In addition, each model has a different ability to encode different language identities. If the typological properties of the language are more consistent with most languages, then that language will be preserved well by the pre-trained multilingual models. We also explored the ability of the models in capturing different typological features, which is generally: morphology > lexicon > word order > simple clause > syntax > verbal category ≈ nominal category. In the layer-wise experiment, the capability of each layer in XLM and XLM-R is more consistent, while mBERT is more susceptible to language and typology, and the performance of each layer fluctuates greatly. Finally, we conducted a case study on Chinese language and found that the abilities of overall model and each layer were significantly lower than the average of sample languages when encoding language identity of Chinese. Also, we found that the model was able to capture lexical and morphological features of Chinese better, but was less effective in predicting the features of syntax, word order and nominal categories. In the future, we will continue to explore how to adopt typological knowledge to eliminate differences between languages, so that the performance of the models can be improved in downstream tasks.

## Supplemental Information

10.7717/peerj-cs.899/supp-1 Supplemental Information 1 The corpus and code

## Additional Information and Declarations

### Data Availability

The following information was supplied regarding data availability: The data is available at Leipzig: https://wortschatz.uni-leipzig.de/en/download. The corpus is available at GitHub:
https://github.com/JianyuZheng/Probing-Language-Identity.
